# Development and Validation of the Father-Love Absence Scale for Adolescents

**DOI:** 10.3390/bs13050435

**Published:** 2023-05-22

**Authors:** Yanhui Xiang, Yue Zhou

**Affiliations:** 1 Teacher Education College, Hunan City University, Yiyang 413000, China; 2 Department of Psychology, Hunan Normal University, Changsha 410081, China; zhoyue@hunnu.edu.cn; 3 Cognition and Human Behavior Key Laboratory of Hunan Province, Hunan Normal University, Changsha 410081, China

**Keywords:** psychological absence, father love, exploratory factor analysis (EFA), confirmatory factor analysis (CFA)

## Abstract

Although father love is vital for the positive growth of the child, there is currently no reliable tool to assess the psychological absence of fathers. Therefore, the current study aims to develop an instrument to measure adolescents’ experiences of father-love absence from a psychological absence perspective. According to the fundamental psychological diathesis assumption, the father-love absence scale (FLAS) was developed based on expert panel discussions. A total of 2592 junior high school student participants were surveyed, and exploratory factor analysis (EFA) and confirmatory factor analysis (CFA) was conducted to determine the items for the formal scale. The results showed that the 18-item FLAS consisted of four factors, which were emotional absence (EA), cognitive absence (CA), behavioral absence (BA), and volitional absence (VA). In conclusion, the FLAS demonstrated satisfactory reliability and validity, and this scale is a valuable tool for assessing father-love absence.

## 1. Introduction

For a long time, the father and mother have been considered the greatest contributors to child development. In the field of family studies, maternal influence on children remains a topic of great interest in psychology, while the role of father love has not been well-studied [1]. In fact, the attachment theory points out that there is no priority between the father–child attachment and the mother–child attachment [2]. Even fathering is unique to individual development, as many studies have shown that the early absence of father love has a wide range of effects on personality, cognitive, emotional, and behavioral development [3,4,5]. Additionally, father love plays an important role in the development of society, and the absence of father love poses a negative impact on society [6]. Although father love is of great importance, the research on father love is still at a rudimentary level under mother-led related development theories [7]. Therefore, the current study is the first to develop a father-love absence scale based on a comprehensive psychological diathesis perspective, in anticipation of laying an essential foundation for building a subsequent theory of father-love absence.

The concept of father-love absence in this article is defined differently from father absence. Specifically, the father absence implies more of a physical absence, while the father-love absence should focus more on the psychological aspect of the absence, such as how many fathers do not give their children a warm father love experience even though living in the family [8]. Furthermore, psychological competence is reflected in the cognitive, emotional, volitional, and behavioral aspects, which constitute the individual’s fundamental psychological qualities [9]. Thus, the present study defines father-love absence as father–child estrangement in childhood regarding emotion, behavior, cognition, and volition. Driven by gender role differences, the father tends to be the financial supporter in the family, and the mother is more often engaged in the daily care and upbringing of the children [10,11]. In this family context, many fathers are not physically absent but are psychologically absent from their kids. Compared to direct physical absence, psychological absence is insidious, which means that most families may not be aware of this widespread and serious phenomenon of father-love absence. Importantly, the experience of father love is necessary for the child’s positive development. A meta-analysis based on 33 studies from 15 countries on four continents found that father indifference or neglect was strongly associated with children’s psychological discomfort and negative personality tendencies such as hostility, emotional instability, and negative self-esteem [5]. According to the fundamental psychological qualities hypothesis and previous empirical evidence, the focus of scale item generation would be on the cognitive, emotional, volitional, and behavioral absence of father love.

Assessing the adolescent experience of father-love absence is crucial since the adolescent is in a critical developmental period characterized by profound physical and psychological changes [12]. Father-love absence adversely affects the physical and mental health of the adolescent. On the one hand, the physiological effect is manifested in the earlier onset of menstruation for women. A large number of cross-sectional and longitudinal research has demonstrated that women who experience father-love absence usually have their menarche one to three months earlier [13,14,15]. On the other hand, several empirical supports have been provided for the effect of father love on psychological development. For example, Le Roux (2009) conducted a cross-cultural study that found that the absence of father love was the most significant predictor of adolescent loneliness. In general, father love shapes many aspects of adolescent development. Additionally, father love has a more prominent role in influencing life satisfaction or happiness than that exerted by the mother [16,17].

Reviewing previous studies, it was found that there has been no research to develop a father love scale based on a psychological absence perspective, and most of the existing father love scales have been constructed using father involvement or presence as a logical beginning. The IFI (inventory of father involvement) is one of the more influential ones. The IFI grew out of Hawkins and Palkovitz’s (1999) rethinking of previous time-based measures of father involvement. They argue that time-based assessment methods are not better measures of the impact of father involvement on their child and that what is more important is the character and content of the involvement. While pointing out the limitations of time-based measurement, Hawkins proposes future directions for father-love assessment (e.g., items should be designed with relatively unique forms of male participation and avoid focusing only on “traditionally female” tasks) [18]. Building on the 1999 review, Hawkins (2002) recruited 723 American fathers aged 22–59 years as participants to formally develop the IFI to advance the multidimensional measurement of father love. The IFI was formulated from the emotional, cognitive, moral, and behavioral aspects of father involvement, and nine factors were identified through exploratory and confirmatory factor analysis. (i.e., discipline and responsibility, school encouragement, mother support, providing, time and talking together, praise and affection, developing talents and future concerns, reading and homework support, attentiveness) [19]. Overall, the 26-item IFI provides a relatively comprehensive understanding of the complex multidimensional structure of father love. Some researchers, however, have noted the limitations of father-reported scales such as the IFI, which suggest that more significant to a child’s current and future development should be the kid’s subjective experience of father love. Put differently, a child perceives that the father is involved in his or her life with high quality. Then, fatherly influence would be the outcome of that perceived involvement, and irrelevant to the child’s perceived authenticity [20]. Moreover, the FPQ (father presence questionnaire), developed by Krampe and Newton (2006) based on a sample of 608 American adults with a mean age of 34.7 years, has also been used widely to some extent. The FPQ was logically constructed from the perspective of father presence as a psychological structure for the child’s understanding of father love, which is reflected in the relationship with the father, beliefs about the father, and intergenerational family influences. Within the three-dimensional framework of father presence, the FPQ generated 10 subscales for measuring individuals’ perceptions and experiences of psychological closeness to their fathers using exploratory and confirmatory factor analysis [21]. Although the FPQ also emphasizes measuring the psychological dimension of the father, considering that the scale was designed for adults, and thus would not accurately capture the fathering experience of the adolescent who is changing more rapidly physically and psychologically. Meanwhile, the FPQ contains many items related to Western religious culture, such as the conceptions of god as father, which are not applicable to families from Eastern cultures.

Encouragingly, in recent years, as sociocultural changes have taken place, both developing and developed countries are increasingly paying attention to the irreplaceable role that father love plays in healthy development among adolescents [22,23]. It is commendable to value father love in family parenting. However, only when the structural factors of father-love absence are elucidated, and reliable and valid measures are developed, can family education and interventions to mitigate the negative effects of father-love absence be improved.

## 2. Materials and Methods

### 2.1. Item Generation

The father-love absence scale items were generated in two ways: (1) For the review of the literature, the literature was searched using the keywords “father presence, father involvement, and child neglect”. Then, the father scale items used in the searched literature were adjusted. (2) The expert panel, which was led by an experienced developmental psychology researcher and several graduates engaged in item-generation discussions, scrutinized the items. This resulted in raw items related to four aspects of father-love absence: emotional absence, cognitive absence, behavioral absence, and volitional absence. To be specific, the 10-item emotional absence refers to the father’s chronic neglect of expressing emotions and understanding the emotional needs of the child. There are 13 items of cognitive absence, referring to the child’s understanding and knowledge of the father’s image. There are 11 items related to behavioral absence, meaning that fathers are rarely engaged in their child’s education and life. Six items assess the volitional absence, which refers to the absence of the father’s influence on the child’s persistence, independence, and decisiveness.

### 2.2. Participants and Procedure

This study was conducted with 2592 (48.9% female) adolescents from two middle schools in central China. The participating adolescents were aged 11 to 16 years (*M* = 13.07, *SD* = 0.90), and included 7th grade (37.5%), 8th grade (30.1%), and 9th grade (32.4%). All participants completed the FLAS by accessing the online platform through the school-organized Youth Potential Growth Assessment program. The present study obtained approval from the authors’ university research ethics committee.

### 2.3. Data Analyses

The data were processed using SPSS 23.0 and Amos 24.0. First, an exploratory factor analysis was conducted using SPSS 23.0 to preliminarily construct a dimensional system of father-love absence among adolescents. Then, a confirmatory factor analysis was performed with Amos 24.0 to validate the theoretical conceptualization and the explored factors. Finally, the construction of the formal scale was established through the two analyses mentioned above.

## 3. Results

### 3.1. Item Analysis

The critical ratio method and correlation analysis were adopted to analyze the items of the father-love absence scale. First, the highest 27% of participants were selected as the high-scores group (total score ≥ 51, *n*_high_ = 707), while the low-scores group consisted of the lowest 27% of participants (total score ≤ 32, n_low_ = 727). Then, the *t*-test was performed on the item scores. The test results are shown in Table 1: the *t*-value for item 40 was not at the significance level (*p* > 0.05), indicating that this item should be deleted for its low differentiation. Meanwhile, the other items of FLAS reached the significance level.

Second, a correlation analysis was conducted between each item and the total score of FLAS. The analysis revealed that, although all items were significantly correlated with the total score, the correlation coefficients for items 7, 15, and 35 were below 0.4, which means that the item is less homogeneous with the whole scale and should be considered for deletion.

Combining the differentiation and correlation results (see Table 1), items 7, 15, 35, and 40 were deleted, and 36 items were retained.

### 3.2. Exploratory Factor Analysis

Since 36 items were retained after item analysis of the father-love absence scale, half of the sample (*n* = 1296) was randomly selected to conduct exploratory factor analysis on the remaining 36 items. Firstly, the necessity calculation is presented in Table 2: Kaiser–Meyer–Olkin index (0.98) and Bartlett’s sphericity test (26331.12, *p <* 0.001, *df* = 630), indicating that common factors existed among the items and were suitable for exploratory factor analysis.

Furthermore, the principal component analysis, without fixing the number of extracted factors, found more than 53% cumulative contribution of variance for the four factors. From the fifth factor, the variance explained by each factor is less than 3%, and the scree plot also shows that scree formation starts at the fifth factor.

Integrating the theoretical assumptions and preliminary exploration, it would be more reasonable to fix the number of factors to 4 for extraction. The criteria for the deletion of items were: (1) factor loading < 0.4; (2) commonality < 0.3; (3) items are loaded high on two or more factors. After deleting those that did not meet the criteria, 29 items remained. Additionally, considering the obvious differences in the number of items across dimensions, and in order to maintain a balanced scale structure, 10 items with low loadings and difficult interpretation in the affective, cognitive, and behavioral dimensions were eliminated. Meanwhile, one item was deleted from the volition factor because it was completely inconsistent with the theoretical assumption. Finally, the 18-item father-love absence scale was composed of 4 factors, with an explained variance of 61.07% and Cronbach’s alpha coefficient of 0.89. The scale items, factor loadings, and reliability coefficients are shown in Table 2.

### 3.3. Confirmatory Factor Analysis

**Figure 1:**
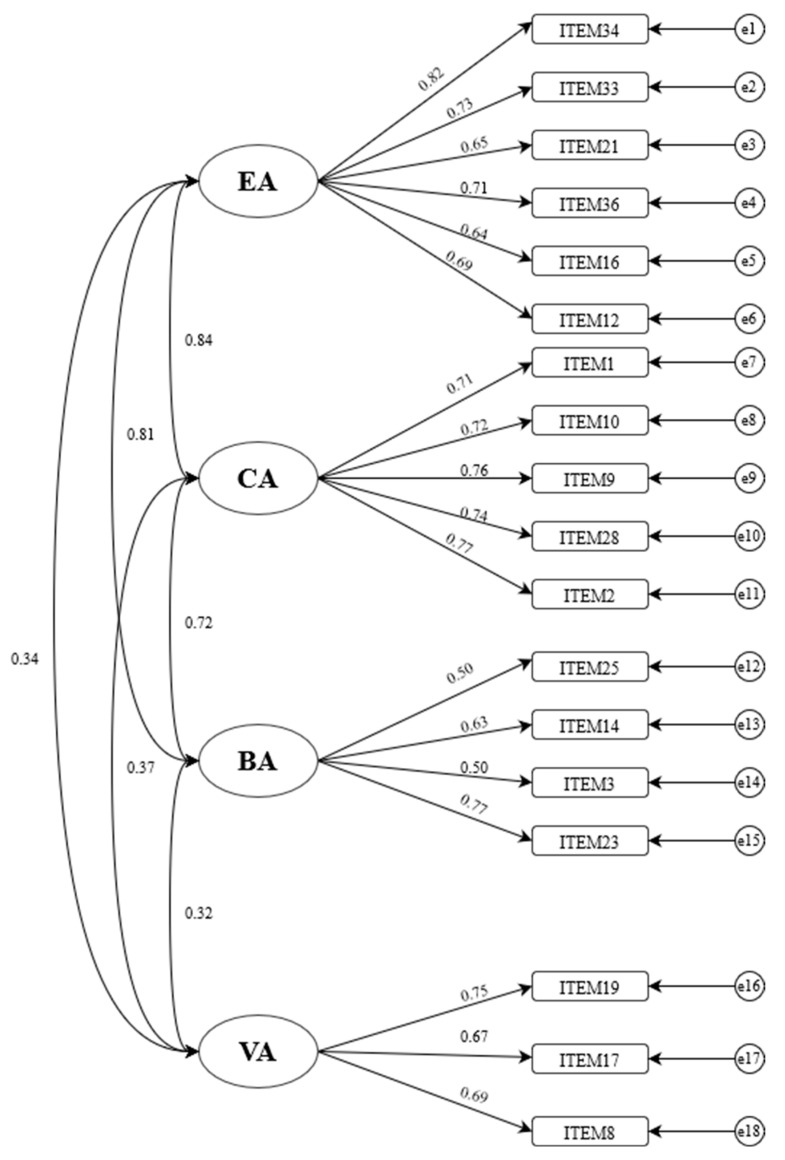
The confirmatory factor analysis model of FLAS. Note. EA = emotional absence; CA = cognitive absence; BA = behavioral absence; VA = volitional absence.

Through the other half of the sample (*n* = 1296), the confirmatory factor analysis was employed to validate the factor structure extracted from the exploratory factor analysis (see Figure 1). According to the criteria suggested by psychometricians, the results show that the model fits well: *χ^2^* = 618.02, df = 129, CFI = 0.95, NNFI = 0.94, RMSEA = 0.05, SRMR = 0.04 [24].

### 3.4. Structural Validity

Correlation analysis was performed between the factors and between the factors and FLAS to examine the structural validity. As shown in Table 3, there was a significant positive correlation between the four factors, indicating a consistent direction among the factors. Additionally, the factors were significantly and positively correlated with FLAS, which suggests that the factors aligned with the general concept.

### 3.5. External Validity

The external validity of the FLAS was assessed by correlating it with two existing father scales that have been widely applied(see Table 4). Two subscales were selected from the FPQ developed by Krampe and Newton (2006), namely feelings about the father and perception of father’s involvement. Additionally, the father version of emotional warmth in the s-EMBU-C (short-Egna Minnenav Barndoms Uppfostran-Chinese), developed by Jiang et al. (2010), was adopted [25].

## 4. Discussion

Based on a multidimensional perspective of psychological absence, current research has developed instruments to measure father-love absence. Combining basic psychological diathesis assumption and exploratory factor analysis, four factors were identified as critical measures of father-love absence, namely cognitive absence, emotional absence, behavioral absence, and volitional absence. In particular, emotional and cognitive absence contributed more to the total score, which may be a positive finding, as previous fathering studies have also more commonly found that fathers’ emotions and children’s perceptions of their fathers are more unique predictors of child development [21,26,27].The four-factor framework for conceptualizing as well as measuring father-love absence is broadly consistent with Hawkins and Palkovitz’s (1999) widely accepted multidimensional structure of father involvement. Meantime, the reliability of each factor meets the requirement of measurement science. The confirmatory factor analysis demonstrated that the model for the father-love absence scale fits well and the loadings of each observable on the latent variables are reasonable. Moreover, the construct validity indicated that the factors were consistent with the general concept, and the external validity analysis also revealed that the father-love absence scale was significantly correlated with the current mainstream father-love measurement instruments. In summary, the results of the analysis provide strong support for the reliability and validity of this scale. Importantly, the current foundational work will not only help to further advance the multidimensional measurement of adolescent experiences of father-love absence but will also support researchers to make further explorations in the fathering field.

The father-love absence scale has a wide range of potential applications. To begin with, this scale could directly enrich the family studies system. While father and mother love are both essential to child development, there are undeniable differences in parenting styles between men and women. For instance, mother-rearing may focus more on interpersonal skills, while father-rearing places more emphasis on moral discipline. A wealth of empirical research has also indicated that father and mother love has unique effects on child development [28,29,30]. Recently, despite the enthusiasm of family researchers for father involvement, it remains relatively weak compared to maternal love studies [27]. Consequently, it is urgent and necessary to systematically examine the role of fathers in the family with psychological absence as a breakthrough point. Contemporary fathers seem to be more involved in child-rearing than those of previous generations, but that increase is negligible in comparison to mother involvement [31]. It is important, then, to quantitatively examine the phenomenon of widowed parenting that currently exists in most families. Evidently, the father-love absence scale could serve as an effective approach to answering the numerous theoretical and practical questions about fathers in the above family studies. Secondly, FLAS is available for family therapy. Most existing theories of child development have the mother–child relationship as a central feature. Under the influence of mainstream mother theories, most of the existing child and family interventions have also been conducted with mothers as the default audience [7]. Undoubtedly, therapists would gain new insight and access from fathers following the incorporation of instruments to assess experiences of father-love absence into family interventions.

While this study has made some contributions to the theory and practice, there are several limitations. First, the data were collected using self-reporting methods, which may lead to social bias effects. Second, the participants were all from China, and the results need to be examined in other cultural contexts. Third, since the instrument was developed for adolescents, it should be validated in the future with elementary and senior high school students.

## 5. Conclusions

In summary, the FLAS developed in the present study is a stable and reliable measure of father-love absence. In the context of the call for fathers to be involved in collaborative parenting, the FLAQ could serve as a tool to support research in this important field.

## Figures and Tables

**Table 1:** The independent sample *t*-test and Pearson correlation analysis.

Item	*t*	*r*_i-T_	Item	*t*	*r*_i-T_	Item	*t*	*r*_i-T_	Item	*t*	*r*_i-T_
1	29.50 ***	0.64 ***	11	46.43 ***	0.70 ***	21	31.18 ***	0.62 ***	31	24.63 ***	0.48 ***
2	39.62 ***	0.68 ***	12	37.44 ***	0.66 ***	22	38.80 ***	0.68 ***	32	37.08 ***	0.62 ***
3	29.40 ***	0.52 ***	13	28.20 ***	0.59 ***	23	48.77 ***	0.69 ***	33	39.11 ***	0.64 ***
4	33.27 ***	0.61 ***	14	33.43 ***	0.55 ***	24	24.91 ***	0.53 ***	34	54.93 ***	0.74 ***
5	43.03 ***	0.68 ***	15	15.76 ***	0.34 ***	25	27.35 ***	0.49 ***	35	16.30 ***	0.35 ***
6	42.26 ***	0.69 ***	16	34.29 ***	0.62 ***	26	32.98 ***	0.62 ***	36	35.47 ***	0.65 ***
7	9.97 ***	0.18 ***	17	19.08 ***	0.40 ***	27	54.67 ***	0.74 ***	37	52.12 ***	0.75 ***
8	33.51 ***	0.52 ***	18	29.50 ***	0.53 ***	28	40.97 ***	0.70 ***	38	33.06 ***	0.67 ***
9	47.58 ***	0.70 ***	19	25.28 **	0.46 ***	29	46.48 ***	0.67 ***	39	22.78 ***	0.42 ***
10	27.64 ***	0.65 ***	20	32.97 ***	0.65 ***	30	28.90 ***	0.57 ***	40	1.47	−0.04

Note. *r*_i-T_ = item-total correlation. ** *p* < 0.01, *** *p* < 0.001.

**Table 2:** Factor loadings for the 18-item Father-Love Absence Scale.

Item Wording	Factor Loading				Commonality
	Factor1	Factor2	Factor3	Factor4	
Q 34	0.82				0.70
Q 33	0.77				0.54
Q 21	0.73				0.60
Q 36	0.72				0.60
Q 16	0.68				0.52
Q 12	0.67				0.52
Q 1		0.89			0.73
Q 10		0.81			0.67
Q 9		0.63			0.63
Q 28		0.62			0.59
Q 2		0.59			0.60
Q 25			0.76		0.57
Q 14			0.73		0.58
Q 3			0.63		0.49
Q 23			0.60		0.64
Q 19				0.85	0.69
Q 17				0.84	0.68
Q 8				0.77	0.67
Cronbach’s alpha (FLAS:0.89)	0.85	0.85	0.70	0.75	
The eigenvalue	6.92	1.79	1.19	1.10	
% of variance	38.45%	9.93%	6.60%	6.08%	

Note. Factor1: emotional absence; Factor2: cognitive absence; Factor3: behavioral absence; Factor4: volitional absence.

**Table 3:** Correlation between the dimensions of FLAS.

	EA	CA	BA	VA	FLAS
EA	1				
CA	0.73 ***	1			
BA	0.61 ***	0.57 ***	1		
VA	0.26 ***	0.29 ***	0.22 ***	1	
FLAS	0.87 ***	0.86 ***	0.80 ***	0.52 ***	1

Note. EA = emotional absence; CA = cognitive absence; BA = behavioral absence; VA = volitional absence; FLAS = father-love absence scale. *** *p <* 0.001

**Table 4:** Correlation between FLAS, FPQ, and EMBU.

	EA	CA	BA	VA	FLAS
FPQ-FF	0.71 ***	0.75 ***	0.52 ***	0.39 ***	0.78 ***
FPQ-PFI	0.63 ***	0.60 ***	0.69 ***	0.34 ***	0.75 ***
EMBU- EW	0.75 ***	0.67 ***	0.60 ***	0.38 ***	0.75 ***

Note. EA=emotional absence; CA = cognitive absence; BA = behavioral absence; VA = volitional absence; FLAS = father-love absence scale; FPQ = father presence questionnaire; EMBU = Egna Minnenav Barndoms Uppfostran. *** *p <* 0.001.

## Data Availability

The data in this study are available from the corresponding authors upon reasonable request.

## References

[1] Cabrera N.J. (2020). Father Involvement, Father-Child Relationship, and Attachment in the Early Years. Attach. Hum. Dev..

[2] Ainsworth M.S. (1989). Attachments beyond Infancy. Am. Psychol..

[3] Le Roux A. (2009). The Relationship between Adolescents’ Attitudes toward Their Fathers and Loneliness: A Cross-Cultural Study. J. Child Fam. Stud..

[4] Carlson M.J., Corcoran M.E. (2001). Family Structure and Children’s Behavioral and Cognitive Outcomes. J. Marriage Fam..

[5] Khaleque A. (2015). Perceived Parental Neglect, and Children’s Psychological Maladjustment, and Negative Personality Dispositions: A Meta-Analysis of Multi-Cultural Studies. J. Child Fam. Stud..

[6] Popenoe D. (1996). Witkout. Wilson Q..

[7] Li X., Meier J. (2017). Father Love and Mother Love: Contributions of Parental Acceptance to Children’s Psychological Adjustment. J. Fam. Theory Rev..

[8] Trivedi S., Bose K. (2020). Fatherhood and Roles of Father in Children’s Upbringing in Botswana: Fathers’ Perspectives. J. Fam. Stud..

[9] Hong L. (2004). Review on Diathesis, Mental Diathesis and Quality Education. Stud. Psychol. Behav..

[10] Craig L., Mullan K. (2011). How Mothers and Fathers Share Childcare: A Cross-National Time-Use Comparison. Am. Sociol. Rev..

[11] Shek D.T.L. (2006). Chinese Family Research. J. Fam. Issues.

[12] Culpin I., Heron J., Araya R., Melotti R., Lewis G., Joinson C. (2014). Father Absence and Timing of Menarche in Adolescent Girls from a UK Cohort: The Mediating Role of Maternal Depression and Major Financial Problems. J. Adolesc..

[13] Belsky J., Steinberg L.D., Houts R.M., Friedman S.L., Dehart G., Cauffman E., Roisman G.I., Halpern-Felsher B.L., Susman E. (2007). Family Rearing Antecedents of Pubertal Timing. Child Dev..

[14] Bogaert A.F. (2008). Menarche and Father Absence in a National Probability Sample. J. Biosoc. Sci..

[15] Moffitt T.E., Caspi A., Belsky J., Silva P.A. (1992). Childhood Experience and the Onset of Menarche: A Test of a Sociobiological Model. Child Dev..

[16] Flouri E., Buchanan A. (2002). Life Satisfaction in Teenage Boys: The Moderating Role of Father Involvement and Bullying. Aggress. Behav..

[17] Flouri E. (2003). The Role of Father Involvement and Mother Involvement in Adolescents’ Psychological Well-Being. Br. J. Soc. Work.

[18] Hawkins A., Palkovitz R. (1999). Beyond Ticks and Clicks: The Need for More Diverse and Broader Conceptualizations and Measures of Father Involvement. J. Men’s Stud..

[19] Hawkins A., Bradford K., Palkovitz R., Christiansen S., Day R., Call V. (2002). The Inventory of Father Involvement: A Pilot Study of a New Measure of Father Involvement. J. Men’s Stud..

[20] Finley G.E., Schwartz S.J. (2004). The Father Involvement and Nurturant Fathering Scales: Retrospective Measures for Adolescent and Adult Children. Educ. Psychol. Meas..

[21] Krampe E., Newton R. (2006). The Father Presence Questionnaire: A New Measure of the Subjective Experience of Being Fathered. Father. A J. Theory Res. Pract. About Men Father..

[22] Leon S.C., Jhe Bai G., Fuller A.K. (2016). Father Involvement in Child Welfare: Associations with Changes in Externalizing Behavior. Child Abus. Negl..

[23] Li X. (2020). Fathers’ Involvement in Chinese Societies: Increasing Presence, Uneven Progress. Child Dev. Perspect..

[24] Marsh H.W., Hau K.T., Wen Z. (2004). In Search of Golden Rules: Comment on Hypothesis-Testing Approaches to Setting Cutoff Values for Fit Indexes and Dangers in Overgeneralizing Hu and Bentler’s (1999) Findings. Struct. Equ. Model..

[25] Jiang J., Lu Z., Jiang B., Xu Y. (2010). Revision of the Short-Form Egna Minnenav Barndoms Uppfostran for Chinese. Psychol. Dev. Educ..

[26] Gerhardt M., Feng X., Wu Q., Hooper E.G., Ku S., Chan M.H. (2019). A Naturalistic Study of Parental Emotion Socialization: Unique Contributions of Fathers. J. Fam. Psychol..

[27] Bi S., Haak E.A., Gilbert L.R., El-Sheikh M., Keller P.S. (2018). Father Attachment, Father Emotion Expression, and Children’s Attachment to Fathers: The Role of Marital Conflict. J. Fam. Psychol..

[28] Hastings P.D., McShane K.E., Parker R., Ladha F. (2007). Ready to Make Nice: Parental Socialization of Young Sons’ and Daughters’ Prosocial Behaviors with Peers. J. Genet. Psychol..

[29] Stolz H.E., Barber B.K., Olsen J.A. (2005). Toward Disentangling Fathering and Mothering: An Assessment of Relative Importance. J. Marriage Fam..

[30] Day R.D., Padilla-Walker L.M. (2009). Mother and Father Connectedness and Involvement During Early Adolescence. J. Fam. Psychol..

[31] McBride B.A., Dyer W.J., Liu Y., Brown G.L., Hong S. (2009). The Differential Impact of Early Father and Mother Involvement on Later Student Achievement. J. Educ. Psychol..

